# Structure–Activity Relationships of Cationic Lipidoids against *Escherichia coli*

**DOI:** 10.3390/antibiotics12081300

**Published:** 2023-08-09

**Authors:** James Jennings, Dunja Ašćerić, Nermina Malanovic, Georg Pabst

**Affiliations:** 1 Institute of Molecular Biosciences, University of Graz, NAWI Graz, 8010 Graz, Austria; 2 Field of Excellence BioHealth, University of Graz, 8010 Graz, Austria

**Keywords:** combinatorial screening, lipidoid, membrane permeabilization

## Abstract

Membrane-active molecules provide a promising strategy to target and kill pathogenic bacteria. Understanding how specific molecular features drive interactions with membrane components and subsequently cause disruption that leads to antimicrobial activity is a crucial step in designing next-generation treatments. Here, we test a library of lipid-like compounds (lipidoids) against Gram-negative bacteria *Escherichia coli* to garner in-depth structure–activity relationships using antimicrobial assays. Modular lipidoid molecules were synthesized in high-throughput, such that we could analyze 104 compounds with variable combinations of hydrophobic tails and cationic headgroups. Antibacterial activity was strongly correlated to specific structural features, including tail hydrophobicity and headgroup charge density, and also to the overall molecular shape and propensity for self-assembly into curved liquid crystalline phases. Dye permeabilization assays showed that *E. coli* membranes were permeabilized by lipidoids, confirming their membrane-active nature. The reduced permeabilization, as compared to Gram-positive *Bacillus subtilis*, alludes to the challenge of permeabilizing the additional outer membrane layer of *E. coli*. The effect of headgroup solubility in gemini-type lipidoids was also demonstrated, revealing that a headgroup with a more hydrophilic spacer between amine groups had enhanced activity against *B. subtilis* but not *E. coli*. This provides insight into features enabling outer membrane penetration and governing selectivity between bacterial species.

## 1. Introduction

Molecules that can act against the cellular envelopes of microbial species offer one promising route to compounds that combat the rise in antimicrobial resistance. At the forefront of research in this field are antimicrobial peptides (AMPs) [1,2,3], some of which belong innately to the defense systems of numerous organisms [4]. Research in the last three decades has shown how interactions with bacterial membrane lipids are often key to AMP modes of action [5,6]. Such membrane interactions are enabled by a combination of non-covalent forces, particularly electrostatic interactions between positively charged residues and the enrichment of anionic lipids in bacterial membranes, van der Waals interactions between hydrophobic residues and lipid hydrocarbon tails, and entropic contributions. These interactions then lead to bacterial cell death through different mechanisms, ranging from the introduction of small pores to wholescale membrane dissolution. The database of studied natural AMPs now exceeds 4000 [7,8], and from the understanding of structure–property relationships, new synthetic AMPs have been designed [1]. In particular, features such as charge and hydrophobicity emerge as key governing features for antimicrobial activity [1]. However, one major drawback of synthetic AMPs lies in the high cost to prepare the compounds on a large scale, thus limiting their potential broad applicability.

To overcome these limitations, alternative membrane-targeting antimicrobials with features resembling AMPs can be prepared by chemical synthesis. In general, amphiphilic compounds comprising cationic charges and sufficient hydrophobic volume have the potential for disruption of bacterial membranes. Thus, quaternary ammonium compounds (QACs) or “quats” find broad use as disinfectants and antiseptics [9,10,11,12,13,14]. However, these compounds also exhibit high toxicity to mammalian cells and are, therefore, not amenable in all areas of application. We recently introduced a new class of lipid-like molecules that are active against Gram-positive *B. Subtilis*, and whose activity strongly depends on several aspects of the molecular structure [15]. Compared to conventional QAC molecules, a multitude of positive charges within the headgroup and a higher number of hydrophobic tails (>4) led to lethal antibacterial activity at concentrations as low as 2 μM. Lipidoid molecules were observed to possess diverse conformations, including (i) chair-like structures, in which the tail groups bridge across two bilayers and the headgroup spans the hydrophilic domain to form a lamellar phase [16]; (ii) disc-like conformations in which the tail groups point out in all directions from the headgroup, resulting in stacking into inverse bicontinuous cubic or inverse hexagonal LCs [17]; and (iii) inverted conical shapes, observed in some lipids with small headgroups or other amphiphilic species with a large hydrophobic volume [15,16]. In addition to lipidoid chemical structures, these conformations are dependent on the aqueous conditions, particularly the nature of the positive charge in the headgroup (introduced by protonation or methylation) and the counterion. 

It has been observed for numerous AMPs and QACs that antimicrobial activity is higher against Gram-positive than Gram-negative bacteria [18,19]. This behavior is typically attributed to the increased challenge of disrupting or penetrating two membranes compared to one. Understanding the differences in activity against the two classes of bacteria is crucial when designing compounds with selectivity for one species over another. In addition, selectivity for bacteria over mammalian cell membranes is essential to the development of next-generation compounds. The specific interactions with membranes are undoubtedly linked to differences in the lipid compositions of membranes from different species. In particular, the lipopolysaccharides (LPS) that reside in the outer leaflet of the outer membrane of Gram-negative bacteria are distinct lipids that produce a barrier that can be difficult to penetrate. Molecules that can bind and neutralize LPS are sought for antimicrobial and antiseptic properties [20]. Gram-positive bacteria, meanwhile, possess lipoteichoic acid (LTA) molecules at the outermost layer embedded within the thick peptidoglycan wall [18,21]. However, studies with AMPs have shown that activity is not significantly hindered by the thick peptidoglycan layer and LTA, and activity can even be enhanced by the polyanionic LTA structure [18]. 

Combinatorial and high-throughput methods allow for structure–property relationships to be studied using large libraries of compounds, from which molecular design rules can be obtained. Such studies have already been applied to AMP design [2,3]. Therefore, modular synthetic systems such as lipidoids, which are prepared from readily-available building blocks [22,23], are well-suited to screening for antimicrobial properties. Structurally diverse QACs have previously been studied but typically comprise species with a fixed number of charged headgroups and tails, particularly gemini structures with two cationic headgroups and two tails [9,10,11,12,13,14,24]. Here, using a combination of 8 polyamine headgroups and 13 acrylate tails, we screen a total of 104 distinct lipidoid compounds for their activity against Gram-negative *E. coli*. Compared to our previous study against Gram-positive *B. subtilis*, *E. coli* was less susceptible to almost every lipidoid structure. Cell permeabilization by lipidoids was studied using propidium iodide assays, and finally, the impact of lipidoid headgroup hydrophilicity on activity against *E. coli* and *B. subtilis* was investigated. This study expands knowledge of molecular features that enable cell membrane disruption of Gram-negative bacteria and how these compare to interactions against Gram-positive membranes.

## 2. Results

### 2.1. Combinatorial Antimicrobial Activity Screening

**Figure 1:**
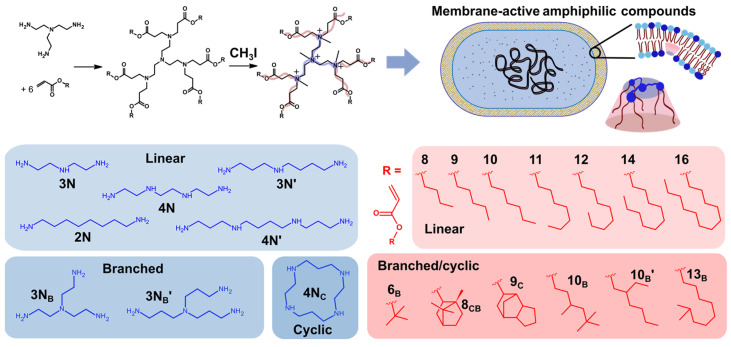
Synthetic route to cationic lipidoids and the polyamines (blue) and acrylates (red) used to synthesize 104 distinct compounds. Headgroup components are labelled xN_y_, where x is the number of reactive amines, and y refers to the architecture (B for branched or C for cyclic). Tails are labelled M_z_, where M is the number of atoms from end to end, and z describes the architecture (B for branched, C for cyclic, or CB for tails with both cyclic and branched features). Where two structures have the same label, a prime is used to distinguish between them. Every amine group reacts with an acrylate through Michael addition to afford amphiphilic species with 4–6 tails. A schematic representation of a lipidoid with a conical shape is shown alongside its proposed mechanism of interaction with an *E. coli* membrane.

Lipidoids were synthesized as described previously [17] from a combination of 8 headgroups and 13 tails, to yield a total of 104 different compounds (Figure 1). The lipidoids varied in their headgroup size (i.e., number of amines and spacer length) and architecture (linear, branched, or cyclic arrangement of amines) and their tail length and architecture (including linear, branched, and cyclic hydrocarbons). These combinations of head and tail groups were previously demonstrated to result in distinct molecular conformations of lipidoids, leading to different liquid crystalline self-assembly behaviors [15,16,17]. All 104 lipidoids were tested against *E. coli* in concentration-dependent assays that allowed rapid assessment of the relative activity of the compounds. Following methylation of the headgroups using methyliodide, the lipidoids were dispersed in the growth medium Muller-Hinton Broth (MHB) and added to bacterial suspensions at 10^6^ CFU/mL. These mixtures were then incubated at 37 °C and growth was measured on a Bioscreen instrument by following the increase in optical density at 480–560 nm (OD_480–560_). Compounds were considered to have significant antibacterial properties either if no growth was observed during the measurement or if growth was delayed by a significant time, which indicated that 99.9% of bacteria had been inhibited or prevented from growing by the lipidoids (see Experimental section for details). 

**Figure 2:**
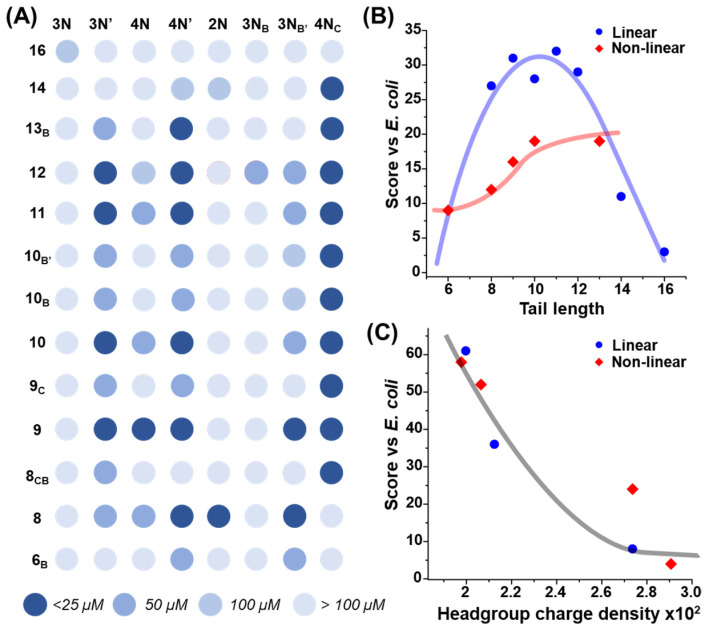
(**A**) Results of combinatorial antimicrobial screening of all 104 lipidoids. Spots are colored according to the lowest concentration at which compounds were active, between 25 and 100 μM, with darker spots indicating lower concentrations. These data were summarized using a scoring system and plotted against different quantitative descriptors of lipidoids with (**B**) tail groups of different lengths and (**C**) headgroups with variable charge density. Lipidoid structures are separated into linear (blue circles) and non-linear (red diamonds) series. Colored lines are added to guide the eye.

The initial broad screen was conducted in the presence of lipidoids at concentrations of 25, 50, and 100 μM to rapidly assess the most effective compounds and identify broad structure–property relationships. The results of this screen are summarized in Figure 2A. These data demonstrated a wide variety of antimicrobial behaviors across the library of lipidoids. Overall, approximately half of the lipidoids were inactive, even at the highest tested concentration of 100 μM. Notably, almost every lipidoid comprising the cyclic **4N_C_** showed antibacterial activity at 25 μM. Across all the other headgroups, variable activity was observed for different tail groups. A scoring system was applied as devised in our prior study to obtain a more quantitative assessment from the large volume of antibacterial assay data [15]. Briefly, this involved awarding points to each lipidoid based on the concentration at which it was active before totaling the scores for each head or tail group (see Experimental section for further details). Every head and tail group was then assigned different quantitative descriptors, including clog*P*, hydrocarbon length, etc. (see Experimental section for complete lists). Scores assigned to given head or tail groups were then plotted against each of these descriptors to screen for global correlations. From these analyses, a notable parabolic relationship was observed between tail length and activity for lipidoids with linear tails (Figure 2B). In addition, a strong negative correlation was observed between headgroup charge density and antibacterial activity (Figure 2C).

The activity against *E. coli* was significantly lower than observed against *B. subtilis* for the same chemical structures. In another study, we observed that 51 lipidoids were active towards *B. subtilis* at 25 μM [15], whereas against *E. coli*, only 24 lipidoids were active at the same concentration. *E. coli* assays were performed in MHB, whereas for *B. subtilis* the optimal growth medium was Luria broth (LB). Therefore, control experiments were performed to test the antibacterial activity of a number of lipidoids against *E. coli* in LB medium, to investigate whether the nature of the medium could contribute to the vastly different activities of lipidoids against the two species. In principle, the medium could affect the lipidoid solubility or aggregation behavior, in a way that might affect its activity. Assays were performed using 10 structurally diverse lipidoids that had demonstrated activity at low concentrations against *B. subtilis* but not against *E. coli* (**3N11**, **4N12**, **3N′9_C_**, **4N′6_B_**, **4N′14**, **3N_B_8_CB_**, **3N_B_10**, **3N_B_10_B_**, **3N_B_′10_B_′** and **4N_C_8**). In every case, lipidoids showed the equivalent antimicrobial activity (or lack of) against *E. coli* at all concentrations studied in both LB and MHB media. Thus, the growth medium was ruled out as a factor affecting the activity of lipidoids against different classes of bacteria.

**Figure 3:**
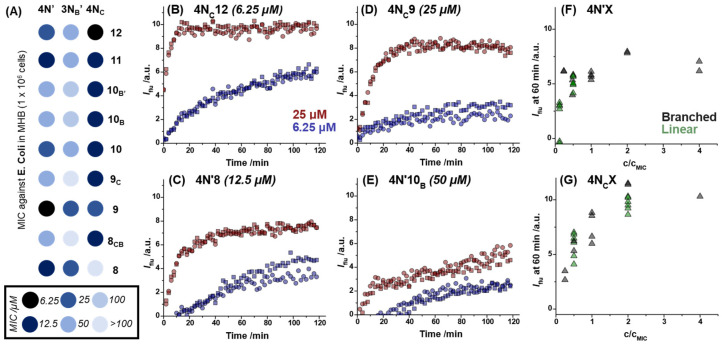
(**A**) Results of lower concentration antimicrobial activity screening, with darker colors signifying activity at lower concentrations. (**B**–**E**) PI permeabilization assay results for four representative lipidoids with different activities (MIC values are noted for each lipidoid in parentheses). Fluorescence intensity (*I*_flu_) was observed to change more rapidly over time in the presence of the lipidoids with lower MIC values. Plots of *I*_flu_ measured after 1 h against the ratio of lipidoid concentration used in the experiment relative to its MIC (c/c_MIC_) for lipidoids with (**F**) **4N′** or (**G**) **4N_C_** headgroups.

A selection of lipidoids were taken forward for antimicrobial assays at lower concentrations for further comparison to activity against *B. subtilis*, for which antibacterial activity was observed as low as 2 μM [15]. Lipidoids with **4N′**, **3N_B_′**, and **4N_C_** headgroups and tails 8–12 atoms long (linear and non-linear) were studied at lower concentrations to identify a lower limit of activity. Results from these minimum-inhibitory concentration (MIC) assays are summarized in Figure 3A. Two lipidoids, **4N′9** and **4N_C_12**, were active down to 6.25 μM. Between the three headgroups, **4N_C_** was consistently the most active, with most showing a MIC at 12.5 μM. Three **4N′** lipidoids were also active at 6.25–12.5 μM, whereas the lowest active concentration for **3N_B_′** lipidoids was 25 μM (for two lipidoids).

### 2.2. Dye Permeabilization Assay

To understand how the antimicrobial activity of lipidoids correlated with membrane disruption, dye permeabilization assays were performed. In our previous study, we investigated the membrane-targeting mechanisms of lipidoids using propidium iodide (PI), a DNA-binding membrane-impermeable dye. This assay can be used to demonstrate when a membrane becomes compromised to such an extent that PI is able to access the cytoplasm. The same experiments were conducted here using *E. coli* in the presence of different concentrations of lipidoid (6.25 and 25 μM) and compared to control experiments in the absence of lipidoid. The permeabilization was followed kinetically by tracking the change in fluorescence in situ during incubation of bacteria with lipidoids in PBS. Control experiments showed no increase in fluorescence, as expected. Intensity data from the control experiments was subtracted from data with lipidoids at each time point to obtain the normalized fluorescence intensity, *I*_flu_, and the kinetics plotted as in Figure 3B–E. The fluorescence data could be further analyzed by comparing the normalized *I*_flu_ values for lipidoids at 60 min. For a selection of lipidoids with **4N′** and **4N_C_** headgroups, these values were plotted against the concentration of lipidoid in the experiment relative to its measured MIC (i.e., c/c_MIC_). These plots demonstrated that cell permeabilization efficiency is key to antimicrobial activity (Figure 3F,G). The higher the concentration of lipidoid relative to its active concentration, the higher the fluorescence increase after 1 h, thus signifying a greater extent of cell membrane damage. In addition, the extent of permeabilization appeared to be higher for **4N_C_** lipidoids than **4N′** at equivalent c/c_MIC_ values, suggesting that the lipidoids with cyclic headgroups are most effective at causing membrane damage. Notably, these absolute fluorescence values were almost an order of magnitude lower than for *B. subtilis*, which will be discussed later in the manuscript. For some lipidoids, *I*_flu_ reached saturation in <60 min (e.g., Figure 3B), but the observation of a slow increase in *I*_flu_ beyond 1 h (i.e., Figure 3E) alludes to partitioning between membranes in other cases.

### 2.3. The Effect of Headgroup Hydrophilicity on Antimicrobial Activity 

**Figure 4:**
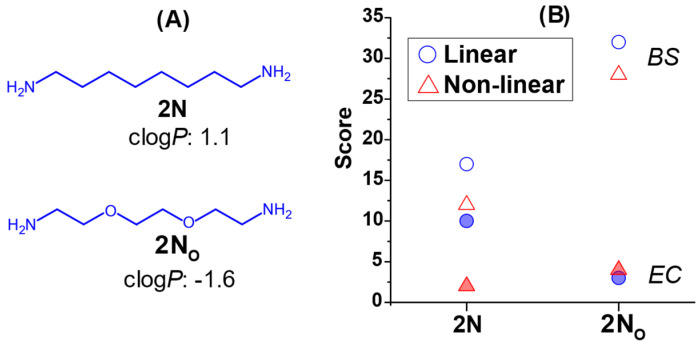
(**A**) Structures of diamines used for lipidoid headgroups with relatively hydrophobic (**2N**) or hydrophilic (**2N_O_**) spacers. (**B**) Comparative scores for lipidoids with **2N** and **2N_O_** headgroups and linear and non-linear tails against *E. coli* (*EC*, filled symbols) and *B. subtilis* (*BS*, open symbols).

Every lipidoid studied here and in our prior study possessed a headgroup with hydrophobic spacers, i.e., hydrocarbons, between the amines. In combination with a multiplicity of hydrocarbon tails, this leads to lipidoids that are typically insoluble in aqueous media, even following headgroup methylation. As a result, methylated lipidoids tended to aggregate into particles that often possess liquid crystalline nanostructure [15]. It is well-known that antimicrobial drug hydrophobicity is one of the greatest barriers to bioavailability [25]. This problem may relate to the challenge of penetrating the bacterial membrane and wall to access particular targets. In order to further assess the effect of lipidoid hydrophobicity and solubility, an additional series of lipidoids were synthesized, which possess a relatively hydrophilic headgroup. Hydrophilicity was introduced using an ethylene glycol diamine headgroup which contains two oxygens (**2N_O_**, Figure 4A). This could be directly compared to the hydrophobic **2N**, which has an identical spacer length (8 atoms long) but is comprised of only methylene groups. **2N_O_**-based lipidoids were synthesized with all 13 tail groups, and their activity was tested against both *E. coli* and *B. subtilis* at concentrations from 25–100 μM. Results from these assays were globally aggregated using the scoring system described earlier. 

Scores against the two species of bacteria were plotted as a function of the headgroup and separated into series of linear or non-linear tails (Figure 4B). Overall, it was clear that increasing the hydrophilicity significantly enhanced the antibacterial activity of both linear and non-linear-tailed lipidoids against *B. subtilis*. Meanwhile, against *E. coli*, hydrophilic headgroups led to much lower activity for lipidoids with linear tails, and only a minor increase in activity for those with branched tails. The introduction of hydrophilicity evidently made an impact on the ability of lipidoids to interact with and/or penetrate bacterial membranes, dependent on the species being targeted. 

## 3. Discussion

Screening the antibacterial behavior of 104 different lipidoids against Gram-negative *E. coli* provides insight into fundamental principles that allow compounds to disrupt cell membranes. As shown in Figure 2B, and in our study against *B. subtilis* [15], tail length is a crucial factor in antimicrobial behavior. Very short tails showed low activity (e.g., **6_B_**), probably due to low overall hydrophobicity that prevents the compounds from inserting into the membranes and causing disruption. Meanwhile, the longer linear tails (**14** and **16**) also displayed significantly lower activity. Notably, these tail lengths are on the order of length of lipid tails. In particular, the outer membranes of *E. coli*, which have typical hydrophobic thicknesses of 24–25 Å [26], coincide with the hydrophobic thickness measured in lamellar phases formed by lipidoids with **14** and **16** tails [17]. Therefore, we hypothesize that matching the hydrophobic thickness of membrane lipids may lead to the effective incorporation of lipidoids but minimal disruption, resulting in longer-tail lipidoids displaying lower antimicrobial properties. Overall, an intermediate tail length (8–12) could create the optimal hydrophobic mismatch that results in maximal membrane disruption. Notably, the branched tail lipidoids performed much worse than the lipidoids with linear tails of equivalent length and hydrophobicity (quantified by the calculated octanol-water partition coefficient, clog*P* [27]). This contrasts with data against *B. subtilis*, which showed that antibacterial activity more closely correlated to clog*P*, independent of tail architecture. The origin of these differences may relate to the different lipid structures between the two species. In particular, *B. subtilis* membranes are known to comprise a majority of branched lipids with tails of 14–17 carbons [28], which branched tail lipidoids could more easily interact with and disrupt. 

Meanwhile, the relationship between activity and headgroup charge density demonstrated that simply increasing positive charge within lipidoids does not enhance activity: the spacing between the charged groups appears to be a more important factor. As our previous study showed, the conformations accessible to lipidoids were key to their activity, and those able to adopt inverted conical shapes were effective against *B. subtilis* at lower concentrations. Such molecules typically included those with longer spacer groups. Results here are consistent with the prior findings, and the lipidoids active against *E. coli* at the lowest concentrations (**4N′9** and **4N_C_12**) were both previously observed to self-assemble into inverted hexagonal phases [15]. Thus, it appears that molecular conformations that impart high negative curvature between hydrophilic and hydrophobic regions are also required to effectively disrupt *E. coli* cell membranes.

Overall, lipidoids were found to be less active against *E. coli* relative to *B. subtilis*, with higher MICs recorded for almost every structure. For the 27 lipidoids studied at lower concentrations, most lipidoids were active against *E. coli* at 2–12.5 times higher concentrations than against *B. subtilis*. This finding follows what has previously been observed for a range of AMPs and QACs when comparing activity against Gram-negative and Gram-positive bacteria. In combination with the data obtained from PI permeabilization assays, which showed much lower fluorescence increases for *E. coli*, data here provide evidence that lower activity against Gram-negative bacteria originates from the difficulty in permeabilizing the two membranes of Gram-negative bacteria versus one found in Gram-positive. The main exceptions to this trend were **4N′12**, **3N_B_′11**, and **3N_B_′12**, which were active against both bacterial species at the same concentrations. Several long-tailed lipidoids from the initial screen (Figure 2A) also showed activity at equivalent or lower concentrations against *E. coli* than *B. subtilis* (including **3N16**, **3N′13_B_**, and **4N′13_B_**). The success of these compounds indicates that higher tail hydrophobicity is optimal against Gram-negative bacteria, which somewhat contradicts theories that high hydrophobicity hinders translocation across the outer membrane [29]. 

Other components of bacterial membranes should also be considered with respect to the mode of action of membrane-active compounds. In particular, proteins embedded within the cell membrane (including lipoproteins and trans-membrane proteins) serve many structural and functional roles. AMPs that specifically recognize lipoproteins are known [30], and some AMPs have been observed to segregate proteins in such a way that inhibits cell wall synthesis, ultimately leading to cell autolysis [31,32]. Since autolysis has been identified as part of the mechanism of action of OCT against Gram-positive bacteria [33], this could also play a role in lipidoid activity. However, the severe disruption of bacterial membranes that we observe is likely to be the primary mechanism of action.

The importance of solubility of different parts of lipidoid molecules was also revealed when comparing structures with **2N** and **2N_O_** headgroups. Lipidoids with hydrophilic **2N_O_** were less effective against *E. coli*, but more effective against *B. subtilis* than those with hydrophobic **2N** headgroup. The oxygen atoms within the hydrophilic lipidoid head could, for example, bind through hydrogen bonding with the sugar moieties on LPS molecules that reside in the outer membrane of Gram-negative bacteria, leading to trapping at the outermost surface. Meanwhile the observed higher activity in *B. subtilis* could originate from enhanced bioavailability, since it was observed that these more hydrophilic lipidoids were often soluble in the growth medium based on the absence of cloudy solutions that indicate the presence of large aggregates. We previously uncovered the importance of molecular conformations on membrane-targeting antibacterial properties, but these latter results also highlight how hydrophobic/hydrophilic balance can also be used as a tool to control interactions with different cell membranes. It is unlikely that the gemini-type surfactants with four tails and hydrophilic headgroups would possess the necessary molecular conformations required to form the inverse hexagonal phase that has been associated with high activity. Therefore, in these cases, the headgroup solubility becomes a more critical parameter. These findings demonstrate once again that the unique conformations of multi-tailed lipidoids set them apart from more commonly studied QACs, which warrants much further study into the details of their mechanism of action. 

## 4. Materials and Methods

Tert-butyl acrylate (**6_B_**, 98%, Sigma-Aldrich, Vienna, Austria), n-butyl acrylate (**8**, 99%, Sigma-Aldrich), pentyl acrylate (**9**, 95%, abcr, Karlsruhe, Germany), isobornyl acrylate (**8_CB_**, 85%, Alfa Aesar, Kandel, Germany), dicyclopentanyl acrylate (**9_C_**, 95%, TCI Deutschland GmbH, Eschborn, Germany), hexyl acrylate (**10**, 98%, Sigma-Aldrich), 3,5,5-trimethylhexyl acrylate (**10_B_**, technical grade, Sigma-Aldrich), 2-ethylhexyl acrylate (**10_B_′**, 98%, Sigma-Aldrich), heptyl acrylate (**11**, 96%, abcr), octyl acrylate (**12**, 98%, TCI), isodecyl acrylate (**13_B_**, TCI) decyl acrylate (**14**, 95%, abcr), dodecyl acrylate (**16**, 90%, Sigma-Aldrich), 1,8-diaminooctane (**2N**, 98%, Sigma-Aldrich), tetraethylene triamine (**3N**, 99%, Sigma-Aldrich), spermidine (**3N′**, 99%, Sigma-Aldrich), hexaethylene tetraamine (**4N**, 97%, Sigma-Aldrich), spermine (**4N′**, 99%, Sigma-Aldrich), tris(2-aminoethyl)amine (**3N_B_**, 96%, Sigma-Aldrich), tris(2-aminopropyl)amine (**3N_B_′**, 97%, TCI), 1,4,8,11-tetraazacyclotetradecane (**4N_C_**, 98%, Sigma-Aldrich) 2,2-(Ethylenedioxy)bis(ethylamine) (**2N_O_**, 98%, Sigma-Aldrich) and ethanol (99.8%, Carl Roth, Karlsruhe, Germany) were all used as received. LB and MHB medium and LB and MHB agar powders were purchased from Carl Roth and diluted with milliQ water obtained from an ELGA Purelab flex system. PBS (e.g., 50 mL in milliQ water) was prepared from 0.38 g NaCl (130 mM), 0.117 g Na_2_HPO_4_ (water-free), and 0.025 g NaH_2_PO_4_ monohydrate, and adjusted to pH 7.4 for PI assays. Octenidine was supplied by Schülke & Mayr (Vienna, Austria).

### 4.1. Lipidoid Synthesis

Reactions were conducted in ethanol at 3.0 M with respect to the total number of moles of amine and acrylate. The acrylate was added in a 1.1 molar excess relative to the total number of reactive amine sites (N). For example, TREN contains three primary amines, which can each react with two acrylates (N = 6), therefore the molar ratio of acrylate to TREN was 6.6:1. Reactions were conducted in 4 mL glass vials in ethanol at 37 °C for 96 h, whilst shaking at 200 rpm in a ThermoFischer MaxQ4450 Fischer Scientific GmbH, Vienna, Austria). Following the reaction, most of the ethanol was removed on a Techne Driblock DB200/3 sample concentrator (Cole-Parmer, Vernon Hills, IL, USA) under a flow of nitrogen at 40 °C. The remaining solvent and unreacted acrylates were removed under vacuum at 65 °C overnight. To enable the high throughput screening approach, we conducted no further purification steps.

### 4.2. Analysis of Purity

**Figure 5:**
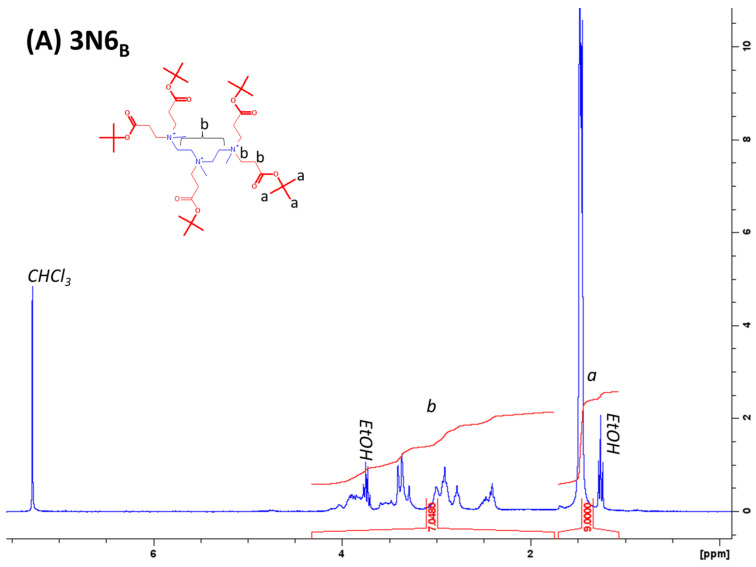

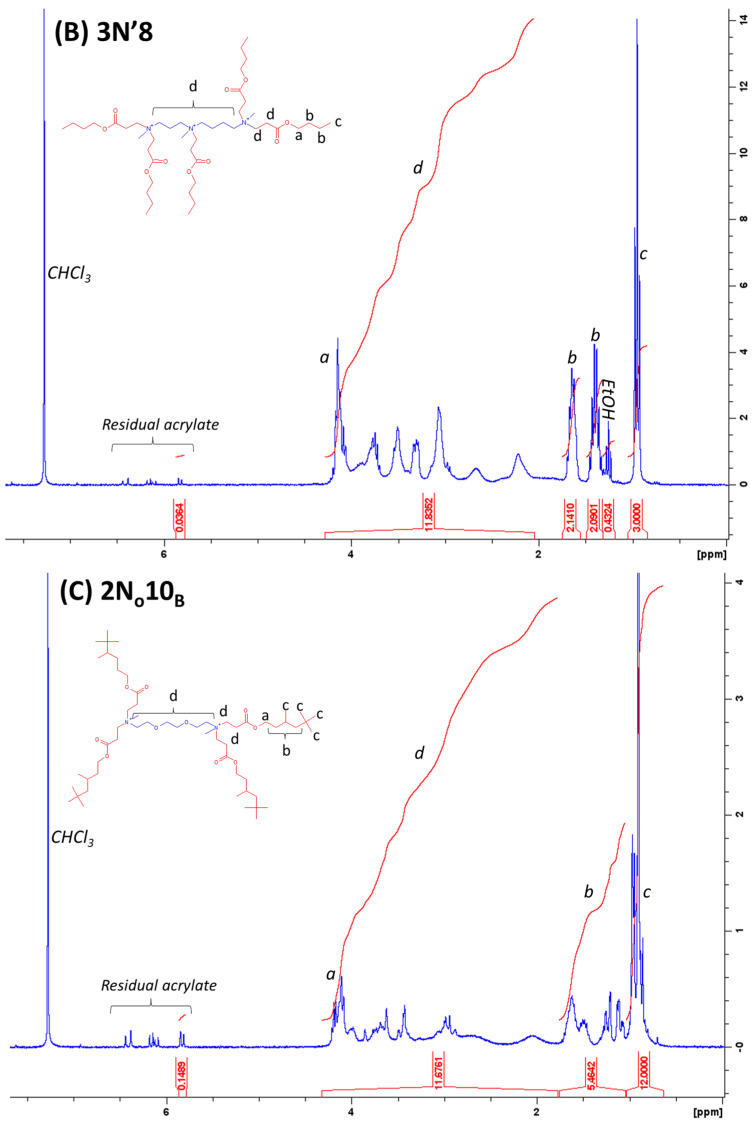

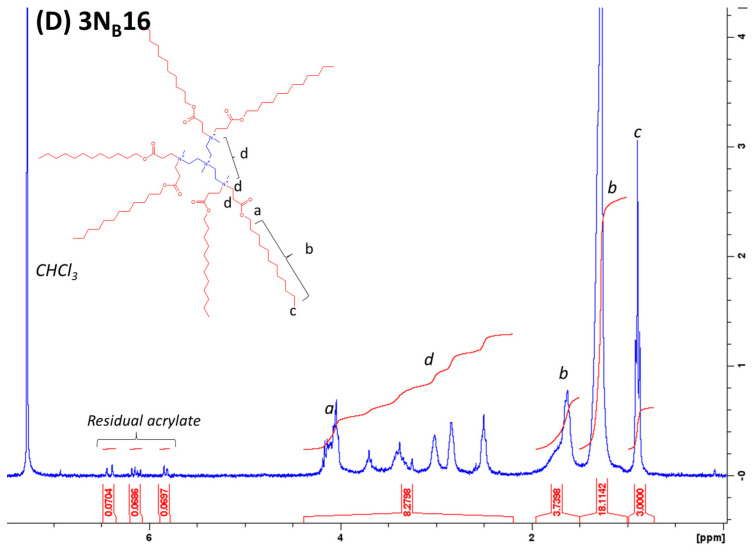
^1^H NMR spectra from methylated lipidoids collected in CDCl_3_. (**A**) **3N6_B_**, (**B**) **3N′8**, (**C**) **2N_O_10_B_**, and (**D**) **3N_B_16**. Peaks assigned to each different proton environments are labeled with lower case latters, and integrations of different regions of the spectra are shown in red. The degree of methylation was calculated from the ratio of *d*, which includes all headgroup protons, including the added methyl groups, to *a*. Degrees of methylation were calculated as 2.5, 3, 4, and 4, respectively.

^1^H NMR spectroscopy was performed to quantify purity using a Bruker Avance III 300 MHz spectrometer with a BBO probehead. Spectra were recorded from an average of 16 scans and analyzed using Bruker TopSpin 4.1.4. Chemical shifts were referenced to the CDCl_3_ peak (7.26 ppm). The details of purity characterization and chemical shifts of peaks observed for all lipidoids are listed in prior studies [15,17]. Examples of ^1^H NMR spectra from methylated lipidoids are displayed in Figure 5.

### 4.3. Bacterial Culture Preparation

WT *E. coli* ATCC25992 or *B. subtilis* 168 trpC2 18 were grown to the mid-log phase and frozen as a stock in glycerol/water, 9/1. This *E. coli* is an FDA-approved strain for susceptibility testing of antimicrobial compounds, with the full-length LPS, and is therefore comparable to biologically relevant resistant strains. *B. subtilis* has been used as a model organism for Gram-positive bacteria for over a centuary, and we note that this strain demonstrated the same susceptibility to OCT as also observed for FDA-approved Gram-positive bacteria *Enterococcus hirae*. However, the cytoplasmic membrane composition of *B. subtilis* is more similar to *E. coli* and is therefore more suitable for comparison of effects besides the cytoplasmic membrane (see [33] and references in there). An aliquot of frozen stock was streaked out onto Agar plates prepared with either MHB (for *E. coli*) or LB (for *B. subtilis*) medium and grown overnight at 37 °C. A single colony from this plate was then transferred to medium (3 mL) and grown for 16–18 h to prepare the overnight culture (ONC). Cell density of the ONC was measured by optical density at 420–580 nm (OD_420–580_) with an ONDA V-10 plus spectrophotometer. An aliquot of the ONC was diluted to an OD value of 0.05 and grown to the mid-log phase (3.5–4 h at 37 °C) for the main culture (MC). The OD_420–580_ of the MC was measured, and 1 mL was pelleted by centrifugation (3400 rpm for 5 min) and washed with PBS buffer twice. Following redispersion in medium or PBS, the main culture was diluted to the desired density (calculated based on an OD_420–580_ = 1 equivalent to 8.8 × 10^6^ cells/mL for *E. coli* and 8.8 × 10^7^ cells/mL for *B. subtilis*). To each well of a 100-well honeycomb plate, 90 µL of the main culture was added. For MIC assays, 1 × 10^6^ cells/mL in LB were employed, whilst for PI permeation assays, a density of 1 × 10^7^ cells/mL in PBS was adopted.

### 4.4. Lipidoid Preparation for MIC Assays

Neat lipidoid (ca. 40 mg) weighed into a tared 2 mL vial was dried under vacuum at 65 °C overnight to remove leftover acrylate, then dissolved in ethanol (0.4 mL). To this solution, a 2.2-fold excess of methyliodide (with respect to total moles of amines) was added, and the solution was left overnight at 22 °C to enable complete methylation. An aliquot of this ethanolic solution (40 μL) was then added to DMSO (0.5 mL) in a 1.5 mL Eppendorf tube. Ethanol and excess methyliodide were removed under nitrogen at 40 °C for 1 h using Techne Driblock DB200/3 (Cole-Parmer, Vernon Hills, IL, USA). The concentration in DMSO was calculated based on the lipidoid mass remaining after vacuum treatment and the remaining volume of DMSO measured by micropipette (ca. 0.45–0.47 mL following evaporation). DMSO solution was added to the MHB or LB medium as required to prepare a 1 mL solution at 1.0 mM. Aliquots of this solution were diluted with MHB or LB medium to 10× the desired value for the MIC assay. A 10 μL aliquot of each concentration of lipidoid dispersion was then added to a well in a honeycomb plate and diluted with bacterial stock (90 μL) to afford a final lipidoid concentration of either 100, 50, 25, 12.5, 6.25, 3, 2, or 1 μM. Each concentration was studied in triplicate, and in cases where results differed between the three wells, the majority outcome from the three experiments was chosen as the MIC value. In each individual assay, a positive control (*E. coli* in MHB medium at 1 × 10^6^ cells/mL) and negative control (*E. coli* with 0.001% *w*/*v* OCT) were also run in triplicate. 

### 4.5. Bacterial Growth Calibration Curve

This procedure followed that reported in our previous publications [15,34]. *E. coli* were grown overnight in MHB medium as described above, and the MC was diluted to 1 × 10^7^, 1 × 10^6^, 1 × 10^5^, 1 × 10^4^, and 1 × 10^3^ cells/mL. 100 μL of each cell density was added in triplicate to a 100-well honeycomb plate. The cultures were grown at 37 °C and followed by measuring OD_420–580_ over a period of 24 h in a Bioscreen C MBR (Bioscreen, Turku, Finland). To determine the onset of the log phase for different cell densities, the first linear region of OD increase was fit with linear regression. The time point at which the fitted line intersected with the baseline was then defined as t_onset_. A plot of t_onset_ vs. cell density was fit with a straight line that allowed cell density to be calculated from onset time for growth. Thus, for each MIC assay, the delay of onset time in the presence of lipidoid relative to the positive control experiment (bacteria only) could be used to estimate the percentage of cells inhibited in growth by lipidoids. In these assays, MIC was defined as the concentration of lipidoid at which >99.9% of bacteria were inhibited, which correlated with a delay of >200 min relative to *E. coli* alone, or >150 min relative to *B. subtilis* alone.

### 4.6. Propidium Iodide Permeation Assay

Bacterial cells were grown as described above and, after washing the MC, were diluted to a concentration of 1 × 10^7^ cells/mL using PBS instead of the growth medium. These cellular suspensions were mixed with an aqueous solution of PI to afford a dye concentration of 2.5 μg/mL. Lipidoid dispersions were prepared as described above in PBS, and 10 μL added (in duplicate) to a black Flat-Bottom Nunc™ MicroWell™ 96-Well plate (Thermo Scientific). To each well, 90 μL of bacteria/dye solution was added, such that the final lipidoid concentrations were 25 and 6.25 μM. As a negative control, 10 μL PBS was mixed with 90 μL of bacteria/dye solution.

### 4.7. Antimicrobial Performance Scoring Criteria

Results from the initial MIC screen were used to generate a score for each combination of head and tail groups. Scores were differentiated by whether the lipidoid entirely prevented bacterial growth during the experimental timeframe or whether it delayed growth to an extent that signified 99.9% growth inhibition. Table 1 describes the process used to select scores, and assigned scores are outlined in Table 2 and Table 3. These scores were then plotted against various descriptors as listed in Table 4 and Table 5. Further details are provided in our previous article [15].

## Figures and Tables

**Table 1:** Summary of the scoring criteria for 104 lipidoids in the initial MIC screen study.

Score	Condition
0	No inhibition at any studied concentration
1	Delayed growth at 100 μM only
2	Delayed growth > 200 min at 100 μM only
3	No growth at 100 μM only
4	Delayed growth > 200 min at 50 μM
5	No growth at 50 μM
6	Delayed > 200 min or no growth at 25 μM

**Table 2:** Scores awarded to each lipidoid based on the initial antimicrobial screen against *E. coli*.

	2N_O_	2N	3N	3N′	4N	4N′	3N_B_	3N_B_′	4N_C_	Tail Total
**6_B_**	0	0	0	0	0	4	1	4	0	9
**8**	0	6	0	4	4	6	0	6	1	27
**8_CB_**	2	1	0	5	0	0	0	0	6	12
**9**	0	1	0	6	6	6	0	6	6	31
**9_C_**	0	0	0	5	0	5	0	0	6	16
**10**	1	0	0	6	5	6	0	5	6	28
**10_B_**	1	0	0	5	0	5	0	3	6	19
**10_B_′**	0	0	0	5	1	5	0	2	6	19
**11**	1	0	1	6	5	6	3	5	6	32
**12**	1	1	0	6	2	6	4	4	6	29
**13_B_**	1	1	0	4	1	6	0	1	6	19
**14**	0	2	0	0	0	3	0	0	6	11
**16**	0	0	3	0	0	0	0	0	0	3
Head total	7	12	4	52	24	58	8	36	61	

**Table 3:** Scores awarded to each lipidoid with a **2N_O_** headgroup based on antimicrobial screens against *B. subtilis*.

Tail	Score
**6_B_**	0
**8**	0
**8_CB_**	6
**9**	5
**9_C_**	5
**10**	6
**10_B_**	5
**10_B_′**	6
**11**	6
**12**	6
**13_B_**	6
**14**	3
**16**	6
Head total	60

**Table 4:** Quantifying parameters used for each tail group.

	MW	clog*P*
**6_B_**	128.2	2.02
**8**	128.2	2.39
**8_CB_**	208.3	4.22
**9**	142.2	2.92
**9_C_**	206.3	3.69
**10**	156.2	3.45
**10_B_**	198.3	4.49
**10_B_′**	184.3	4.33
**11**	170.3	3.98
**12**	184.3	4.5
**13_B_**	212.3	5.39
**14**	212.3	5.57
**16**	240.4	6.64

clog*P* values taken from ChemSpider [35], which lists values predicted using ACD/Labs log*P*).

**Table 5:** Quantifying parameters used for each headgroup.

	N/MW ^#^ (Charge Density)	clog*P* *
**2N**	0.0139	1.1
**2N_O_**	0.0135	−1.59
**3N**	0.0291	−1.87
**3N′**	0.0206	−0.84
**4N**	0.0274	−2.18
**4N′**	0.0198	−0.96
**3N_B_**	0.0274	−2.68
**3N_B_′**	0.0212	−1.01
**4N_C_**	0.0200	−0.97

**^#^** Number of nitrogens divided by the molecular weight of the headgroup, * clog*P* values taken from ChemSpider [35], which lists values predicted using ACD/Labs log*P*).

## Data Availability

Data supporting reported results will be provided upon request by the corresponding author.
